# Editorial: Regulation of proteolysis and proteome composition in plant response to environmental stress

**DOI:** 10.3389/fpls.2022.1080083

**Published:** 2022-11-15

**Authors:** Mateusz Labudda, Shaojun Dai, Zhiping Deng, Ling Li

**Affiliations:** ^1^ Department of Biochemistry and Microbiology, Institute of Biology, Warsaw University of Life Sciences-SGGW, Warsaw, Poland; ^2^ China Development Center of Plant Germplasm Resources, College of Life Sciences, Shanghai Normal University, Shanghai, China; ^3^ Institute of Virology and Biotechnology, Zhejiang Academy of Agricultural Sciences, Hangzhou, China; ^4^ Department of Biological Sciences, Mississippi State University, Mississippi State, MS, United States

**Keywords:** abiotic stress, biotic stress, protease, signal transduction, protein post-translational modification

## Introduction

Because of their sedentary lifestyle, plants are susceptible to changing environmental conditions. They must cope with miscellaneous abiotic stresses usually enhanced by heavy industry (Labudda et al., 2022). Moreover, changes in climate conditions and agricultural systems are favourable to pests and pathogens gradation on plants (Nykiel et al., 2022; Skoracka et al., 2022). However, plants are not defenceless; they are equipped with a battery of multiple mechanisms (from molecular through biochemical-physiological to structural) that are activated by them to ensure further growth and development and the production of diasporas (Muszyńska and Labudda, 2019; Formela-Luboińska et al., 2020; Tokarz et al., 2020; Tokarz et al., 2021; Fidler et al., 2022; Miernicka et al., 2022). Among these mechanisms, the control of proteolysis and thereby the quality and composition of proteins and pool of amino acids are of fundamental significance (Muszyńska et al., 2019; Labudda et al., 2020; O’Conner et al., 2021; Pan et al., 2021; Szewińska et al., 2021; Tan et al., 2021; Sun et al., 2022; Tanvir et al., 2022; Xing et al., 2022).

Proteolysis is an elementary biochemical process indispensable for protein metabolism. A wide spectrum of proteolytic enzymes is involved in this process, including exopeptidases (amino and carboxypeptidases) and endopeptidases (serine, aspartic, metallo- and cysteine peptidases) (Godson and van der Hoorn, 2021; van der Hoorn and Klemenčič, 2021). As uncontrolled proteolysis can seriously damage plant cells, the activity of peptidases is accurately regulated by their endogenous inhibitors (Prabucka et al., 2017; Kunert and Pillay, 2022). Thus, an understanding of the mechanisms assuring the accurate regulation of peptidase activity and the dynamic alterations in the proteome and amino acids of plants struggling with environmental stresses is an urgent task undertaken by numerous teams from all over the world.

This Research Topic aimed to widen the understanding on protein and amino acid metabolism mechanisms in plants using an interdisciplinary approach. The Research Topic contains ten papers from several fields across abiotic stress (acid rain, low and elevated temperatures, salt, osmotic, and abscisic acid (ABA) treatments, and phosphate starvation) and biotic stress (infection with *Verticillium dahliae*). Two review articles are a valuable addition to the experimental articles. The first review by Zhang et al. concerns the application of TurboID-based proximity labelling in studying the protein interaction network in plant response to abiotic stress, while the second by Mangena shows the pleiotropic effects of recombinant protease inhibitors in plants.

## What new have we learned from this Research Topic?

As Topic Editors, it was our pleasure and honour to review and manage the submitted manuscripts. In this editorial, we recapitulate the main findings of the published articles.

One of the abiotic stress, acid rain (AR) may cause severe damage to plant functioning. This problem is especially noticeable in woody plants. Hu et al. investigated the response of *Taxus wallichiana* var. *mairei* to AR stress. These authors showed that, in *T. wallichiana* var. *mairei* plants grown under AR stress in the soil with low calcium (Ca) level, the net photosynthetic rate and activity of the superoxide dismutase, ascorbate peroxidase, guaiacol peroxidase, and catalase were decreased in leaves; however, these physiological parameters were enhanced in plants cultivated under high Ca level in the soil. Furthermore, the proteomic profiling revealed forty-four differentially abundant proteins in leaves of AR stress-exposed *T*. *wallichiana* var. *mairei* plants cultivated under different Ca amounts in the soil. Identified proteins were classified into seven groups related to processes such as signal transduction, protein modification and degradation, metabolism, photosynthesis and energy, cell rescue and defence, transcription and translation and unknown proteins.

Another important abiotic stress is low temperature. Liu et al. presented the results concerning the enzyme aminoacyl tRNA synthetase YLC3, which has been shown to take part in the regulation of amino acid homeostasis and chloroplast thylakoid development in *Oryza sativa* plants under low temperature. This article showed a thermo-sensitive rice mutant *yellow leaf chlorosis3* (*ylc3*) with decreased chlorophyll content, changed thylakoid structure, and increased amounts of aspartate, asparagine, and glutamine in leaves under low-temperature stress.

It is well- known that a plant’s response to stress is governed by an intricate network of phytohormones. Among these hormones are auxins. Ma et al. published results on the *Arabidopsis thaliana* endoplasmic reticulum (ER)-localized MAIGO2 (MAG2) complex and its protein homologue MAG2-Like (MAL) as regulators of plant development and vesicle trafficking and auxin homeostasis with functional redundancy. Moreover, it has been proven that MAIGO2 and MAG2-like participated in stress response, and in more detail the salt, osmotic, and ABA treatments have been examined.

Another published article concerned phosphate (Pi) stress. Wang et al. presented changes in phosphorylation and succinylation of *Hordeum vulgare* root proteins in response to phosphate starvation and recovery. The study showed that, the phosphorylated proteins associated with purine, the mitogen-activated protein kinases (MAPKs), pyrimidine, and ATP-binding cassette (ABC) transporters were upregulated in both Pi starved and recovered barley plants. s regards the succinylome, proteins in nitrogen and phenylpropanoid metabolism were significantly upregulated; on the other hand, proteins in lysine and tryptophan metabolism in both Pi-starved and recovered barley plants were significantly downregulated.

Adamiec et al. took a closer look at the role of the *A. thaliana* chloroplast EGY3 pseudoprotease in response to high-light and high-temperature stresses. Based on the molecular, biochemical, and physiological experiments, these authors concluded that, EGY3 mediated plant response to high-light and high-temperature stresses, and its role was related to photosystem I and light-independent reactions of photosynthesis. Moreover, these authors made the conclusion that, EGY3 took part in the regulation of H_2_O_2_ level through stabilization of the copper/zinc superoxide dismutase 2. Therefore, this matched up, as the authors concluded, to retrograde chloroplast-nucleus signalling.

An important global environmental problem is the soil salinity. Chen et al. using the quantitative proteomics analysis investigated changes in cell wall proteins of *Solanum lycopersicum* root in response to salinity. Two *S. lycopersicum* genotypes were used in this experiment, the salt-tolerant IL8-3 and the salt-sensitive M82 plants. This approach allowed the authors to show the differential responses of two contrasting genotypes. The salinity-tolerant IL8-3 plants presented not only a remarkably decremented Na^+^ level but also a clearly improved redox balance and cell wall lignification in response to salinity in comparison to the salt-sensitive M82 *S*. *lycopersicum*. The common response of plants from both lines was that the proteins involved in signal transduction and cell wall polysaccharides were upregulated in response to salt stress.

In addition to abiotic stresses, biotic stresses, including fungal infections, contribute to losses in crops of arable plants. Lu et al. performed a comparative proteomic analysis of two *Gossypium barbadense* cultivars differing in *V. dahliae* tolerance (susceptible XH7 and resistant XH21). It was clear from this study that, changes in reduced ascorbate (AsA) and H_2_O_2_ contents and the gene expression of ascorbate peroxidases (APX) were essential for *V. dahliae* resistance in *G. barbadense*. Compared to susceptible XH7 plants, the resistant XH21 plants presented consonantly higher AsA level and sharply induced the APX gene expression.

An article by Zhang et al. presented proteomic analysis of the ClpX proteolytic complex consisted of a hexameric ATPase ClpX and a tetradecameric peptidase ClpP in the model cyanobacterium *Synechocystis* sp. PCC 6803. One of the used experimental approaches was the comprehensive proteomic identification of proteins which were ClpX-regulated in *Synechocystis*. One hundred seventy-two ClpX-regulated proteins were detected. As the functional analysis showed, these proteins were engaged in glycolysis, nitrogen assimilation, photosynthetic electron transport, ATP-binding cassette (ABC) transporters, and signal transduction.

Taken together, this Research Topic clearly shows that, investigations of various aspects of proteolysis and proteome composition in plant responses to environmental stresses are conducted by numerous teams from all over the world. This topic is timely from the point of view of modern plant biology. Therefore, there is a strong need for further research in this area, not only in the field of responses to environmental stresses, but also in terms of plant growth and development regulation.

## Author contributions

All authors listed have made a substantial, direct, and intellectual contribution to the work and approved it for publication.

## Acknowledgments

Topic Editors deeply thank all the authors and reviewers who have participated in this Research Topic. We would also like to thank Professor Martin Černý from the Mendel University in Brno (Czech Republic), as the Associate Editor, finally edited this editorial.

## Conflict of interest

The authors declare that the research was conducted in the absence of any commercial or financial relationships that could be construed as a potential conflict.

## Publisher’s note

All claims expressed in this article are solely those of the authors and do not necessarily represent those of their affiliated organizations, or those of the publisher, the editors and the reviewers. Any product that may be evaluated in this article, or claim that may be made by its manufacturer, is not guaranteed or endorsed by the publisher.

## References

[B1] Fidler J. Graska J. Gietler M. Nykiel M. Prabucka B. Rybarczyk-Płońska A.. (2022). PYR/PYL/RCAR receptors play a vital role in the abscisic-acid-dependent responses of plants to external or internal stimuli. Cells 11, 1352. doi: 10.3390/cells11081352 35456031 PMC9028234

[B2] Formela-Luboińska M. Chadzinikolau T. Drzewiecka K. Jeleń H. Bocianowski J. Kęsy J.. (2020). The role of sugars in the regulation of the level of endogenous signaling molecules during defense response of yellow lupine to *Fusarium oxysporum* . Int. J. Mol. Sci. 21, 4133. doi: 10.3390/ijms21114133 32531938 PMC7312090

[B3] Godson A. van der Hoorn R. A. L. (2021). The front line of defence: a meta-analysis of apoplastic proteases in plant immunity. J. Exp. Bot. 72, 3381–3394. doi: 10.1093/jxb/eraa602 33462613 PMC8042752

[B4] Kunert K. J. Pillay P. (2022). Loop replacement design: a new way to improve potency of plant cystatins. FEBS J. 289, 1823–1826. doi: 10.1111/febs.16335 34979048

[B5] Labudda M. Dziurka K. Fidler J. Gietler M. Rybarczyk-Płońska A. Nykiel M.. (2022). The alleviation of metal stress nuisance for plants–a review of promising solutions in the face of environmental challenges. Plants 11, 2544. doi: 10.3390/plants11192544 36235410 PMC9571535

[B6] Labudda M. Różańska E. Prabucka B. Muszyńska E. Marecka D. Kozak M.. (2020). Activity profiling of barley vacuolar processing enzymes provides new insights into the plant and cyst nematode interaction. Mol. Plant Pathol. 21, 38–52. doi: 10.1111/mpp.12878 31605455 PMC6913211

[B7] Miernicka K. Tokarz B. Makowski W. Mazur S. Banasiuk R. Tokarz K. M. (2022). The adjustment strategy of venus fytrap photosynthetic apparatus to UV-a radiation. Cells 11, 3030. doi: 10.3390/cells11193030 36230991 PMC9564066

[B8] Muszyńska E. Labudda M. (2019). Dual role of metallic trace elements in stress biology-from negative to beneficial impact on plants. Int. J. Mol. Sci. 20, 3117. doi: 10.3390/ijms20133117 31247908 PMC6651804

[B9] Muszyńska E. Labudda M. Hanus-Fajerska E. (2019). Changes in proteolytic activity and protein carbonylation in shoots of *Alyssum montanum* ecotypes under multi-metal stress. J. Plant Physiol. 232, 61–64. doi: 10.1016/j.jplph.2018.11.013 30537613

[B10] Nykiel M. Gietler M. Fidler J. Prabucka B. Rybarczyk-Płońska A. Graska J.. (2022). Signal transduction in cereal plants struggling with environmental stresses: from perception to response. Plants 11, 1009. doi: 10.3390/plants11081009 35448737 PMC9026486

[B11] O’Conner S. Zheng W. Qi M. Kandel Y. Fuller R. Whitham S. A.. (2021). GmNF-YC4-2 increases protein, exhibits broad disease resistance and expedites maturity in soybean. Int. J. Mol. Sci. 22, 3586. doi: 10.3390/ijms22073586 33808355 PMC8036377

[B12] Pan J. Li Z. Wang Q. Guan Y. Li X. Huangfu Y.. (2021). Phosphoproteomic profiling reveals early salt-responsive mechanisms in two foxtail millet cultivars. Front. Plant Sci. 12. doi: 10.3389/fpls.2021.712257 PMC8488109 34616412

[B13] Prabucka B. Mielecki M. Chojnacka M. Bielawski W. Czarnocki-Cieciura M. Orzechowski S. (2017). Structural and functional characterization of the triticale (× *Triticosecale* wittm.) phytocystatin TrcC-8 and its dimerization-dependent inhibitory activity. Phytochemistry 142, 1–10. doi: 10.1016/j.phytochem.2017.06.008 28654769

[B14] Skoracka A. Laska A. Radwan J. Konczal M. Lewandowski M. Puchalska E.. (2022). Effective specialist or jack of all trades? experimental evolution of a crop pest in fluctuating and stable environments. Evol. Appl. 15, 1639–1652. doi: 10.1111/eva.13360 PMC9624081 36330306

[B15] Sun M. Qiu L. Liu Y. Zhang H. Zhang Y. Qin Y.. (2022). Pto interaction proteins: critical regulators in plant development and stress response. Front. Plant Sci. 13. doi: 10.3389/fpls.2022.774229 PMC8960991 35360329

[B16] Szewińska J. Różańska E. Papierowska E. Labudda M. (2021). Proteolytic and structural changes in rye and triticale roots under aluminum stress. Cells 10, 3046. doi: 10.3390/cells10113046 34831267 PMC8618286

[B17] Tanvir R. Ping W. Sun J. Cain M. Li X. Li L. (2022). *AtQQS* orphan gene and *NtNF-YC4* boost protein accumulation and pest resistance in tobacco (Nicotiana tabacum). Plant Sci. 317, 111198. doi: 10.1016/j.plantsci.2022.111198 35193747

[B18] Tan J. Zhou Z. Feng H. Xing J. Niu Y. Deng Z. (2021). Data-independent acquisition-based proteome and phosphoproteome profiling reveals early protein phosphorylation and dephosphorylation events in arabidopsis seedlings upon cold exposure. Int. J. Mol. Sci. 22, 12856. doi: 10.3390/ijms222312856 34884660 PMC8657928

[B19] Tokarz K. M. Wesołowski W. Tokarz B. Makowski W. Wysocka A. Jędrzejczyk R. J.. (2021). Stem photosynthesis–a key element of grass pea (*Lathyrus sativus* l.) acclimatisation to salinity. Int. J. Mol. Sci. 22, 685. doi: 10.3390/ijms22020685 33445673 PMC7828162

[B20] Tokarz B. Wójtowicz T. Makowski W. Jędrzejczyk R. J. Tokarz K. M. (2020). What is the difference between the response of grass pea (*Lathyrus sativus* l.) to salinity and drought stress?–a physiological study. Agronomy 10, 833. doi: 10.3390/agronomy10060833

[B21] van der Hoorn R. A. L. Klemenčič M. (2021). Plant proteases: from molecular mechanisms to functions in development and immunity. J. Exp. Bot. 72, 3337–3339. doi: 10.1093/jxb/erab129 33847361 PMC8042755

[B22] Xing J. Tan J. Feng H. Zhou Z. Deng M. Luo H.. (2022). Integrative proteome and phosphoproteome profiling of early cold response in maize seedlings. Int. J. Mol. Sci. 23, 6493. doi: 10.3390/ijms23126493 35742945 PMC9224472

